# Estimation of Potential Savings Associated With Switching Medication Formulation

**DOI:** 10.1001/jamahealthforum.2021.4823

**Published:** 2022-02-04

**Authors:** Sunita M. Desai, Jiejie Wang, Uttara M. Ananthakrishnan, Ishita Ghai, Ateev Mehrotra, Hemant K. Bhargava

**Affiliations:** 1 Department of Population Health, New York University School of Medicine, New York; 2 Foster School of Business, University of Washington, Seattle; 3 University of California Davis Graduate School of Management, Davis; 4 Department of Health Care Policy, Harvard Medical School, Boston, Massachusetts; 5 Division of General Medicine, Beth Israel Deaconess Medical Center, Boston, Massachusetts

## Abstract

This cross-sectional study describes the price differences between capsule and tablet or ointment and cream forms of prescription drugs for insured patients.

## Introduction

One underrecognized opportunity to decrease spending for drugs that are available in multiple formulations is to switch to a lower-cost formulation.^1,2^ In this study, we examined medications that are available in pairs of formulations: capsule or tablet and ointment or cream. For each medication, we estimated the savings from shifting to a lower-cost formulation.

## Methods

The New York University School of Medicine Institutional Review Board determined that this cross-sectional study did not involve interaction with human participants, and the data used did not contain protected health information. We followed the Strengthening the Reporting of Observational Studies in Epidemiology (STROBE) reporting guideline.

We analyzed a convenience sample of medication orders for patients with coverage by a large insurance plan across health systems in California, Minnesota, and New Jersey during a 1-month period (August 1-31, 2019). Each medication order contained information on the drug (defined as medication in a specific concentration), formulation, days’ supply, and total price paid for the drug (the sum of the insurer and patient payment). We separately examined both tablet and capsule formulations as well as ointment and cream formulations of the medications. We limited the analyses to drugs in our data with at least 5 orders for each formulation.

We assigned a daily price to each medication-formulation combination by dividing the total price paid by the days’ supply averaged across orders. We calculated the proportion of orders that were placed for the higher-cost formulation according to these daily prices.

To estimate the potential savings from switching formulations, we calculated the minimum potential expenditure for each medication order by multiplying the days’ supply ordered with the per day price assigned to the lower-cost formulation. We estimated the total spending for each order by multiplying the days’ supply ordered with the mean daily price for the ordered formulation. The difference between the total and minimum spending as a percentage of total spending was the opportunity for savings; we summed the opportunity for savings overall and by medication.

## Results

A total of 28 medications available as a tablet or a capsule and 21 medications available in cream or ointment form were analyzed. The sample contained 8368 orders that were initiated for patients.

**Figure 1.**
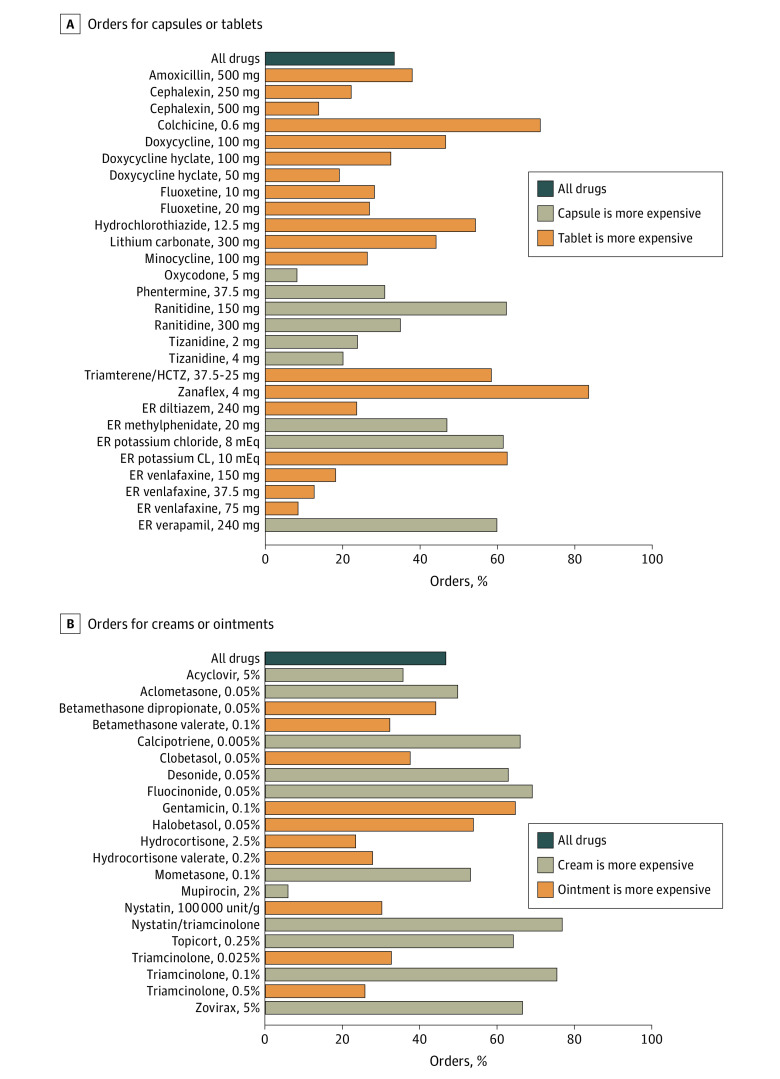
Percentage of Orders for Higher-Cost Formulation by Medication CL indicates chloride; ER, extended release; and HCTZ, hydrochlorothiazide.

**Figure 2.**
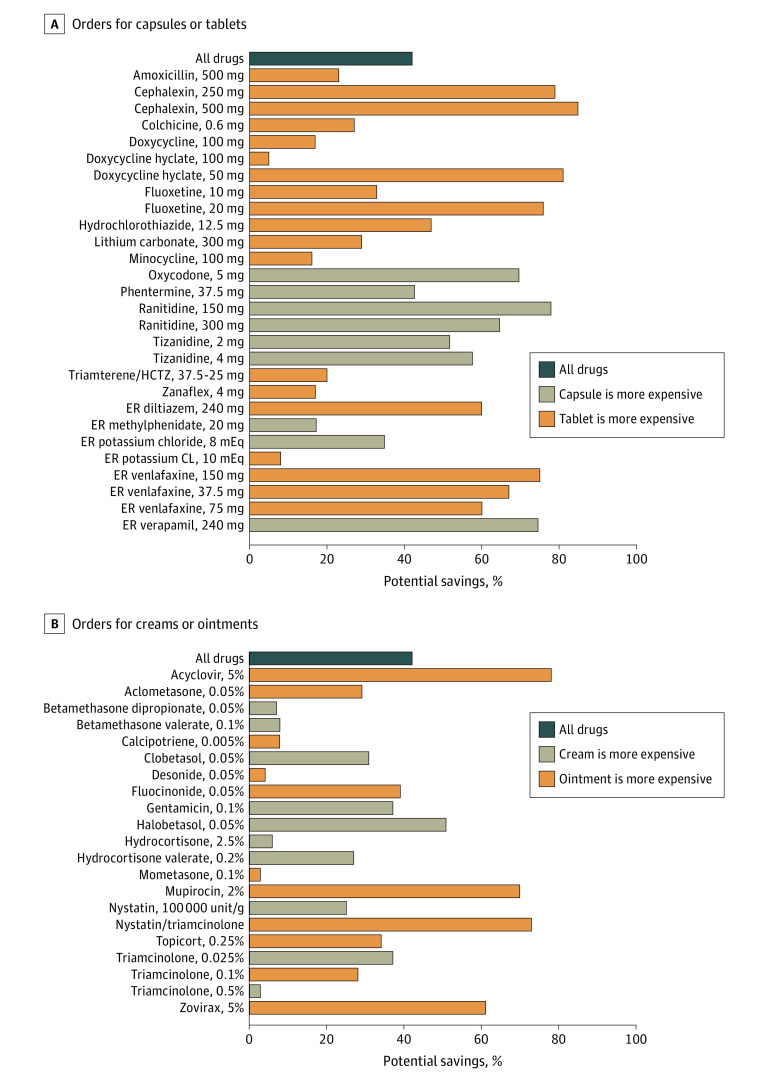
Simulated Opportunity for Savings From Switching to a Lower-Cost Formulation by Medication CL indicates chloride; ER, extended release; and HCTZ, hydrochlorothiazide.

For tablets and capsules, 33% of orders (n = 1255) were placed for the higher-cost formulation (Figure 1). If these orders were instead placed for the lower-cost formulation, aggregate spending across the analyzed drugs would have been reduced by 42% (Figure 2). Nineteen drugs were more expensive in tablet form, and 9 drugs were more expensive in capsule form.

For ointments and creams, 47% of orders (n = 2153) were placed for the higher-cost formulation. If the lower-cost formulation had been ordered, spending on these medications would have been 42% lower. Eleven medications were more expensive in cream form, and 10 drugs were more expensive in ointment form. Across all orders, before the exclusion criteria were imposed, 25% of medications were ordered in more than 1 formulation.

## Discussion

There has been limited recognition of the substantial cost differences in formulations of the same drug. In this study, we found that switching medication formulations can reduce expenditure on the drugs we analyzed by more than 40%. If prescribers were made aware of these differences (for example, through price transparency solutions), they might be able to help patients realize substantial savings. However, given that formulations may not be entirely substitutable,^3,4^ prescribers and patients may need to weigh savings against the differences in convenience or beneficial outcome. Furthermore, savings may be limited because not all drugs are available in multiple formulations.

A limitation of this study was that it examined medication orders for a small population of patients who were covered by the same health insurer. Nonetheless, we believe the study identified formulation as a dimension along which drug prices can vary and described the potential for decreasing drug expenditures.
